# Absent in Melanoma 2 Gene Associated Periodontitis and Coronary Heart Disease

**DOI:** 10.12688/f1000research.151954.2

**Published:** 2025-07-14

**Authors:** Zina Ali Daily, Batool Hassan Al-Ghurabi

**Affiliations:** 1 Periodontics, University of Baghdad, College of Dentistry, Baghdad, Iraq; 2 Periodontics, University of Al-Ameed,College of Dentistry, Karbala, Iraq; 3 Basic Science, University of Baghdad, College of Dentistry, Baghdad,, Iraq

**Keywords:** periodontal disease; coronary heart disease; gene polymorphism; AIM2 gene.

## Abstract

**Aims:**

To study the association between AIM2 gene polymorphisms and the tendency for periodontal infection and coronary heart disease, and to determine whether males or females are more susceptible to these diseases. Additionally, we examined its association with the features of periodontal disease.

**Methods:**

140 patients were enrolled in this study, and those who took part were divided into four groups as follows: healthy (c), periodontal disease (P), coronary heart disease with intact periodontium (AS-C), and coronary heart disease with periodontal disease (AS-P). Information on entrants, including age ranging from 40 to 70 years old, sex of both males and females who fulfilled the study’s criteria, body mass index with ordinary rang less than 30 kg/m ^2^, socioeconomic status, tooth brushing frequency at least two times per- day, and indicators of periodontal disease severity, was documented. Blood samples were collected, and AIM2 gene polymorphisms were evaluated by polymerase chain reaction test, gel phase, and sequences.

**Results:**

Genetic analysis of AIM2 G/T (rs *2793845*) revealed a high frequency of the (T) allele and (GT and TT) genotypes that were detected in the periodontal disease and coronary heart disease groups in males. The Hardy-Weinberg equilibrium of alleles and genotypes did not differ significantly (p > 0.05) between the study groups. Gene polymorphisms were also significantly (p ≤ 0.05) correlated with indicators of periodontal disease severity.

**Conclusion:**

High frequenting of (T) alleles and (GT, TT) genotypes in AIM2 single nucleotide polymorphisms (SNP) were associated with an increased tendency to develop periodontal disease and coronary heart disease. It can be supposed that it has a causative function in the pathophysiology of both disorders, and the validity of SNP as a potential genomic factor for the risk of both disorders in Iraqi males.

## Introduction

The tissue connection and support teeth in position are demolished by an infectious inflammatory illness known as periodontitis. By transitioning from a symbiotic to a dysbiotic condition, pathobionts also harm the tissue. ^1^^,^^2^ Immune responses are mostly controlled by genotypes, and dysbiotic flora also influences the response. The distribution and severity of diseases are influenced by genetic factors, which also have a substantial impact on the immune response to bacterial infections. ^3^ In response to an increase in the levels of lipids and low-density lipoprotein (LDL) in the blood, the immune system launches, propagates, and triggers atheroma throughout the circulatory system, resulting in atherosclerosis (AS), an inflammatory disease. Atherosclerosis is one sign of coronary heart disease (CHD). ^4^^,^^5^ An assembly of several proteins, known as the AIM2 inflammasome, occurs in response to microbiome or sterile allusions. During apoptosis, inflammatory mediators are released into cells as a result of the active form of caspase-1. Moreover, it is linked to osteoclast stimulation, atheroma formation, and progressive periodontal ligament injury. ^6^^–^^8^ Numerous studies have documented a correlation between genetic variation in the inflammasome constituent genomes and heightened tendency toward periodontal disease or CHD among diverse ethnic groups. ^9^^,^^10^ Genetic illnesses, such as autoimmune sickness, dermatitis, and periodontal disease, have also been reported to be associated with variants of the AIM2 genetic factor, known as DNA, which consists of two strands of sensors. ^11^ To date, there is no evidence from any research linking a variant of the AIM2 gene to periodontal disease risk in people with or without CHD. This sheds insight into the current investigation, which aims to determine the relationship between AIM2 gene polymorphisms and periodontal disease propensity in both people with and without CHD, and to detect whether males or females are more susceptible to these diseases. We also evaluated the correlation between AIM2 single nucleotide polymorphisms and indicators of periodontal disease severity.

## Methods

Case-control study design from November 2022 to May 2023, the inquiry occurred in numerous centers in Baghdad City. The study was conducted in accordance with the Declaration of Helsinki, and approved by the Ethics Committee of the University of Baghdad, College of Dentistry (Ref. 652, 13/09/2022, Project # 652622) that gave their stamp of approval to the study’s moral foundations. All the human research protocols complied with the ethical standards laid out in the Helsinki Statement and its later modifications. Each participant signed an informed written consent form after being provided detailed information about the study and its purposes. The present study followed the Strengthening the Reporting of Observational Studies in Epidemiology (STROBE) in terms of design of the study and results reporting.

### Sample

This investigation evaluated 140 entrants, including males and females, aged range (40-70) years, all with an ordinary range of body mass index (BMI), socioeconomic status and tooth brushing frequency at least two times per- day who were recruited in this study according to the inclusion and exclusion criteria. The entrants were diagnosed according to the 2017 classification of periodontal diseases and conditions with severity of cases in periodontal disease groups. ^12^^,^^13^ The standardized indicators of periodontal disease were clinically assessed using a UNC-15 probe for each tooth, including CAL and PPD, which were measured to the nearest millimeter, the whole-mouth plaque index (PLI), ^14^ and whole-mouth BOP. ^15^ The diagnosis was made by a cardiologist specialist for CHD through the coronary arterial catheter technique, presenting current atheroma in excess of 75%. ^16^^–^^19^ The specialist of cardiology is blinding to subjects of CHD patients that related to CHD with periodontitis group or CHD with healthy periodontium group.

Inclusion criteria included individuals (a) through an acceptance to participate in the investigation, (b) in excellent general wellness, other than a diagnosis of atherosclerosis from a heart catheter technique, and without any other systemic illnesses. Elimination standards involved individuals (a) with physical disorders, (b) undergoing periodontal therapy within the previous period of six months, (c) were smoking behavior, (d) had reserved inflammation inhibitor medicine in the previous period of three months; and (e) were either breastfeeding or pregnant when the research was conducted. Following the recruitment of research entrants, each participant signed a declaration of consent. After that, every entrant’s blood sample was taken, clinical evaluations were thoroughly documented, and demographic details were noted.

**Figure 1.**
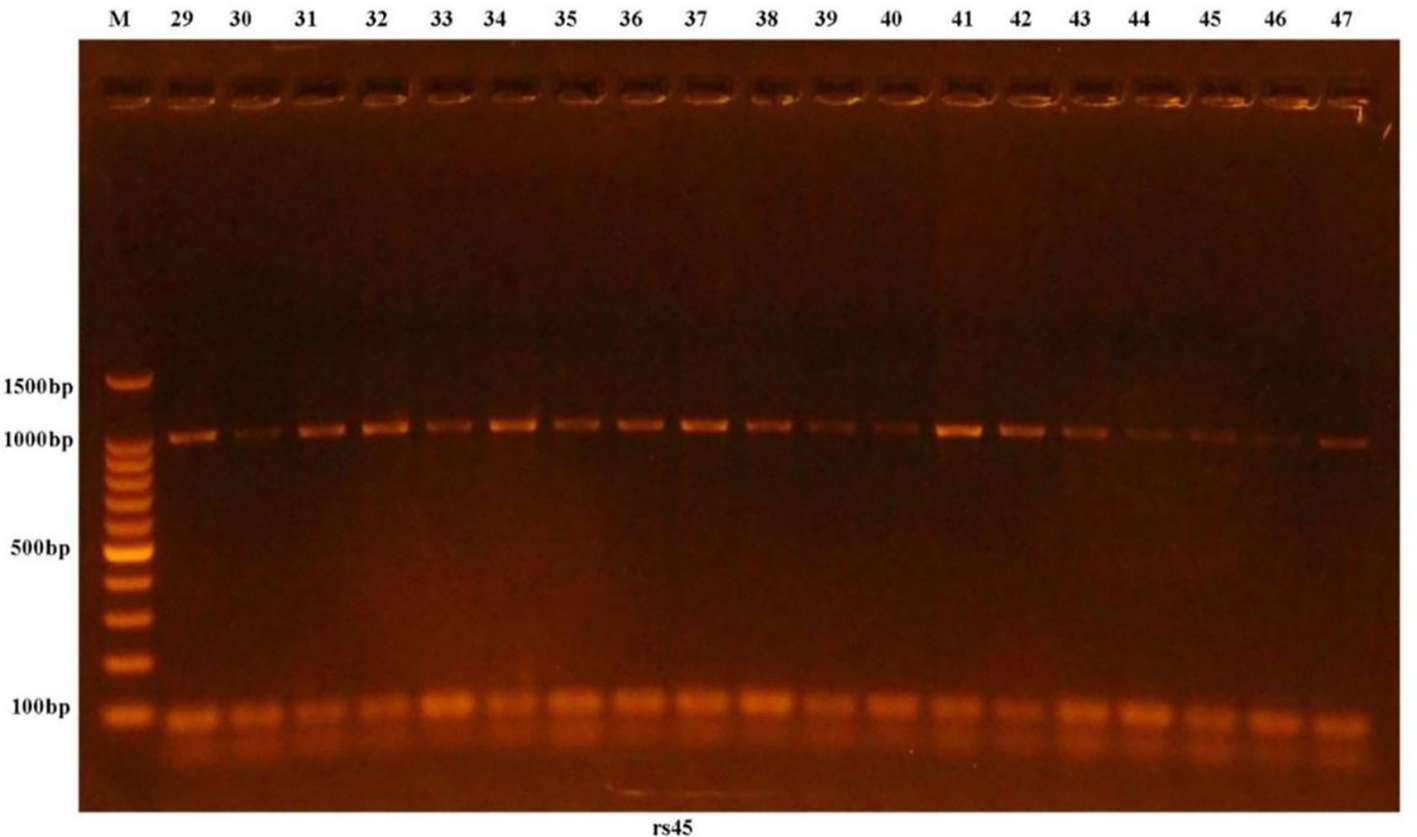
Results of the amplification of rs2793845 in 976 bp of human samples were observed in agarose gel electrophoresis, M: 100 bp ladder marker.

Gathering and examination of blood samples in less than 30 s, 2 mL of venous blood was pulled, placed in an ethylenediaminetetraacetic acid (EDTA) tube, and kept at -40°C for the (AIM2) genome using PCR-sequencing techniques. Genomic DNA was extracted from the blood samples using the ReliaPrep Blood gDNA Miniprep System (Promega, USA) DNA purification kit, and the isolated DNA was assessed using a Quantus Fluorometer. To create a functional primer solution, the lyophilized form was submerged in nuclease-free water (NFW). The appropriate temperature for primer annealing was also determined. The DNA template was multiplied using identical forward and reverse primer pairs at the annealing DNA template. PCR enhancements were then performed. Following PCR augmentation, multiplication was demonstrated using electrophoresis with agarose gel (OWL, Thermo, USA) combined with 10 mg/mL ethidium bromide stain (EBS) (Promega, USA), as shown in Figure 1. The PCR outcomes were analyzed by Sanger sequencing using a Macrogen ABI3730XL Automated Genomic Sequencer (Korea). The blind laboratory doctor collected, process blood samples and detected gene polymorphism. The statistic of data process by blind analyzer.

The accuracy, validity, and reproducibility of the researcher were assessed by carrying out an inter- and intraexaminer calibration; categorical variables (PI and BOP) were assessed by using a kappa-coefficient assay. The targeted level was kappa value >75% to decide a good level of agreement was present. For continuous variables (PPD and CAL), the level of agreement rounded to the nearest millimeter should be >0.9 as determined by interclass coefficient assay. ^20^ An investigation of the same examiner’s validity was necessary to determine the dependability of this study. After assessing five patients with periodontal disease, two calibrating meetings for indicators of periodontal disease severity were obtained within an hour. For PPD and CAL, the intraclass coefficient was 0.96, while BOP and PLI had kappa coefficient assessed 0.85 and 0.94. Consequently, the dependability level of the investigation was adequate.

The main genetic marker of AIM2 in the research groups was identified on the basis of the findings of the pilot study. With an estimated power of 80% and an alpha likelihood ratio of 0.05, an overall number of 140 entrants (35 individuals for each group) were determined for the sample size using Odds/Ratios and https://epitools.ausvet.com.au/casecontrolss.

### Statistical analysis

Graph Pad software Inc. La Jolla, CA’s Prism version 9.0 was used to do the statistical evaluation. ^21^ The normality of distribution was checked by the Shapiro–Wilk and D’agostino’s tests. Categorical variables were expressed as frequency and percentage, while average and standard deviation were used to describe continuous variables. The average and deviations for every of the categories’ separate variable. One-way ANOVA was used to compare differences between continuous data, and determined the significant difference between the examined groups. In addition, categorical variables were analyzed by using the chi-square test. The correlation between indicators of periodontal disease severity and AIM2 polymorphism gene was identified by Pearson correlation coefficients. The significant difference will be revealed if p<0.05. The intergroup differences in the distributions of the SNP was analyzed using the chi-square test. Frequency distributions for selected variables were done first. Hardy–Weinberg equilibrium (HWE) is used to calculate the expected alleles from the observed genotypes. The odds ratio (OR) measures the strength of association between the presence of certain polymorphisms and disease status.

Additionally, the use of multiple logistic regression for analysis the association of AIM2 gene polymorphism with susceptibility to periodontitis and CHD.

## Results

**Figure 2.**
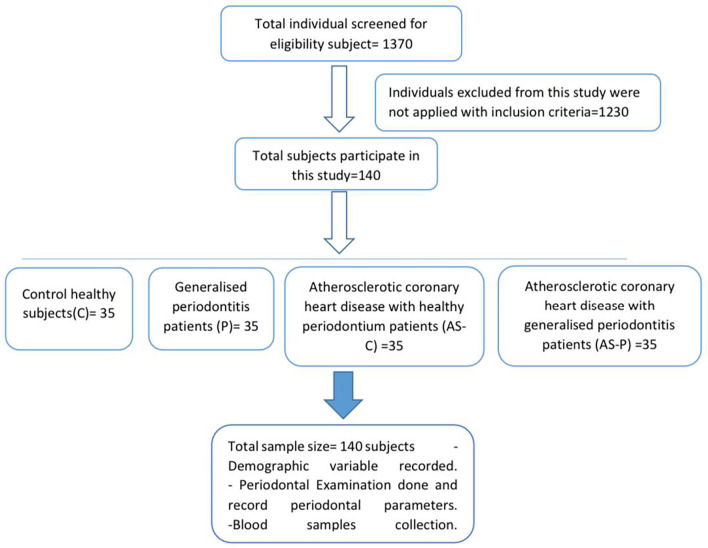
Cardiovascular disease and periodontal disease individuals’ research design schematic.

**Figure 3.**
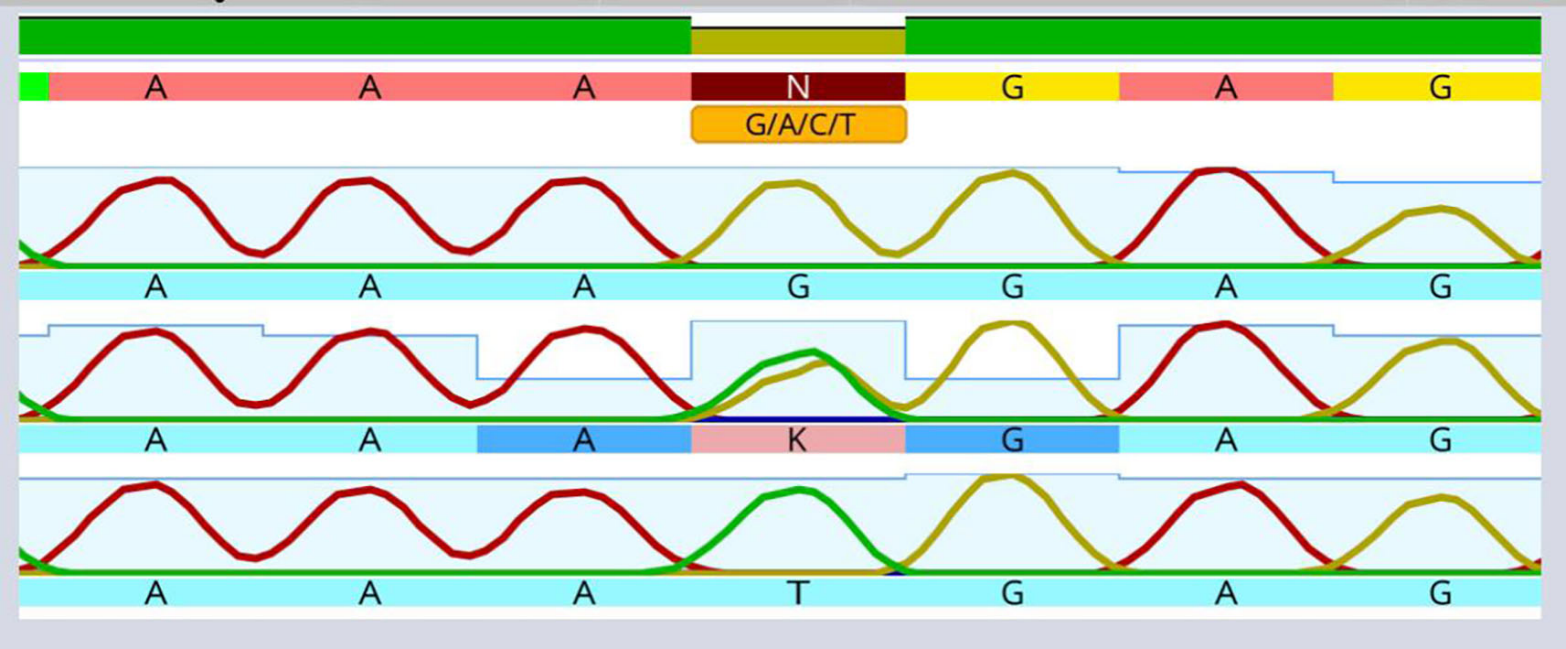
Utilizing Sanger sequencing, the rs *2793845* single nucleotide polymorphism of the AIM2 gene was investigated.

A total of 1370 people examined to participate in the current investigation, whereas 1230 individuals declined based on the grounds for exclusion, as illustrated in Figure 2. The average value was computed considering the demographic factors of the 140 individuals in the four groups (healthy (c), periodontal disease (P), coronary heart disease with intact periodontium (AS-C), and coronary heart disease with periodontal disease (AS-P)). The sex distribution of all groups was 80% males and 20% females. This study examined the BMI of individuals with a weight of 24 kg/m ^2^. Variations in age, sex, and BMI and socioeconomic status across the groups were not statistically significant (p ≤ 0.05) ( Table 1). Moreover, the present outcomes determined a significant increase in the average values of indicators of periodontal disease severity and tooth brushing frequency at least two times per- day in the periodontitis patient groups. Sequencing of the AIM2 SNP using the Sanger method in the four study groups is shown in Figure 3. This SNP, rs *2793845*, results from the replacement of guanine (G) with thymine (T) in the intron region of one chromosome.

**Table 1.** The age, sex, BMI, socioeconomic status, tooth brushing frequency and indicators of periodontal disease in the study groups.

Groups/variables	Control (C)	Periodontitis (P)	CHD and clinically healthy periodontium (AS-C)	CHD with periodontitis (AS-P)	p Value
Age	56.84±9.34	55.12±5.89	57.13±0.34	58.32±9.27	0.061
BMI	24.12±2.69	24.23±0.38	24.97±0.78	24.93±0.81	0.529
Sex					1.00
Male ( *n*, %)	26(80.0%)	26(80.0%)	26(80.0%)	25(76.7%)	
Female ( *n*, %)	9(20.0%)	9(20.0%)	9(20.0%)	10(23.3%)	
Socioeconomic Status					0.312
Low (n, %)	8(22.8%)	9((25.7%)	5(14.2%)	11(31.4%)	
Middle (n, %)	12(34.2%)	20(57.1%)	18(51.4%)	14(40%)	
High (n, %)	15(42.8%)	6(17.1%)	12(43.2%)	10(28.5%)	
Tooth brushing frequency per- day	98(57.0%)	35(40.7%)	73(56.6%)	28(20.7%)	0.052 *
PLI	15.32±1.43	73.063±1.193	20.33±1.89	81.35±3.76	0.001 *
BOP	7.12±3.45	73.28±3.62	7.16±1.22	88.21±4.53	0.001 *
PPD	0.00±0.00	7.32±0.33	0.00±0.00	8.15±0.46	0.001 *
CAL	0.00±0.00	8.19±2.63	0.00±0.00	9.43±0.56	0.001 *

NS = non-significant,

* significant at *p* ≤ 0.05, PLI, plaque index; BOP, bleeding on probing; PPD, probing pocket depth; CAL, clinical attachment loss.

A solitary “T” top point to the presence of a T homozygous for allele. A solitary “G” top point to the presence of a G homozygous for allele. The existence of the “G” and “T” points suggests the existence of the G/T heterozygous allele.

Furthermore, the present study was identified TT genotype of AIM2 G/T (rs *2793845*) was a high odd/ratio (OR=14.9 (3.990 to 45.98), *P*-value=0.0) in P group, (OR=16.43 (0.5094 to 0.4878), *P*-value=0.0013) in AS-C group and (OR=16.45 (0.05094 to 0.4878), *P*-value=0.0013) in AS-P group as compared to control healthy group, whereas genotype GT was a high odd/ratio (OR=22.44 (0.009776 to 0.7495), *P- *value=0.0227) in P group, (OR=9.12 (0.007127 to 0.6108), *P*-value=0.0056) in AS-C group and (OR=6.93 (0.004114 to 0.3088), *P*-value=0.0002) in AS-P group than control healthy group, (p-value<0.000), as described in Table 2. Data analysis of alleles frequencies in patients and controls groups are summarized in Table 3, the T allele of the AIM2 G/T (rs *2793845*) gene showed greater frequenting in the P group was (0.85), AS-C group was (0.72), and AS-P group was (0.8) compared to the G allele, and it is slightly higher than the allele frequency in Asians was 0.65 and the global population was 0.86 from the Genome Aggregation Database (gnom AD). In addition, the frequencies of alleles and genotypes in the AIM2 gene polymorphism did not show any substantial variation relative to HWE in any group of participants. The correlation of AIM2 with indicators of periodontal disease severity in the patient groups is shown in Table 4. There was a significant correlation between AIM2 G/T (rs *2793845*) and BOP (r=0.444, p=0.013; r=0.463, p=0.030), PPD (r=0.364, p=0.048; r=0.399, p=0.029), and CAL (r=0.504, p=0.005; r=0.536, p= 0.004) in the P group and AS-P group, respectively.

**Table 2.** Hardy-Weinberg equilibrium and Distributing of Genotype in AIM2 of The study groups.

Genotypes	C	P	*P*	Chi χ ^2^	RR	OR (95% Cl)	AS-C	*P*	Chi χ ^2^	RR	OR (95% Cl)	AS-P	*P*	Chi χ ^2^	RR	OR (95% Cl)
GG	28 (79%)	2 (4%)	0.000 **	36.27	5.6	85.0 (14.6 to 122)	4 (13%)	0.000 **	29.8	4.6	26 (6.318 to 83.43)	0 (0%)	0.0001 **	37.15	5.76	83.0 (15.20 to 1265)
GT	2 (3%)	9 (23%)	0.0227 *	5.2	0.22	22.44 (0.00 to 0.74)	11 (30%)	0.0056 **	7.7	0.172	9.12 (0.0071 to 0.61)	16 (43%)	0.0002 **	13.42	0.1133	6.93 (0.004 to 0.3088)
TT	5 (17%)	25 (74%)	0.000 **	19.46	3.4	14.9 (3.990 to 45.98)	20 (57%)	0.0013 **	10.33	0.34	16.43 (0.050 to 0.48)	19 (57%)	0.001 **	10.33	0.34	16.45 (0.050 to 0.487)
HWE X ^2^	0.003	0.1726					2.183					3.0				
*p*	0.898 ^NS^	0.853 ^NS^					0.321 ^NS^					0.412 ^NS^				

NS = non-significant,

* significant at *p* ≤ 0.05,

** significant at *p* ≤ 0.01, Chi χ ^2^ = Chi-square, RR = Relative risk, OR = Odds ratio, HWE χ ^2^ = Hardy-Weinberg Equilibrium chi-square.

**Table 3.** Alleles frequenting in AIM2 of the study groups.

Allele	C	P	*P*	Chi χ ^2^	RR	OR (95% Cl)	AS-C	*P*	Chi χ ^2^	RR	OR (95% Cl)	AS-P	*P*	Chi χ ^2^	RR	OR (95% Cl)
G	0.8 (49)	0.15 (11)	0.000 *	53.39	4.76	25.24 (9.70 to 66.69)	0.28 (19)	0.000 *	34.48	3.6	11.27 (4.703 to 26.75)	0.2 (16)	0.000 *	43.25	4.17	16.1 (6.343 to 37.14)
T	0.2 (11)	0.85 (59)					0.72 (43)					0.8 ()				

NS = non-significant,

* significant *p* value ≤ 0.05.

**Table 4.** Pearson correlation test coefficients (r) calculated between AIM2 G/T ( *rs2793845*) and indicators of periodontal disease for the study groups.

SNP		PLI		BOP		PPD		CAL	
	Patients Groups	r	p	r	p	r	p	r	p
AIM2 G/T ( *rs2793845*)	(P group)	0.109	0.565	0.444	0.013 *	0.364	0.048 *	0.504	0.005 *
	(AS-C group)	-0.085	0.655	-0.054	0.776	0.00	0.00	0.00	0.00
	(AS-P group)	0.146	0.443	0.463	0.030 *	0.389	0.029 *	0.536	0.004 *

* Significant at *p* < 0.05; r, Pearson’s coefficient correlation; PLI, Plaque indicator; BOP, bleeding on Probing; PPD, Probing Pocket depth; CAL, clinical attachment loss.

The present findings found significant association (p<0.05) of age, sex, BMI, socioeconomic status, tooth brushing frequency, clinical periodontal parameters, and AIM2 gene polymorphism with susceptibility to periodontitis and CHD. As well as this result revealed a significant association of variables with severity of both conditions, as observed in Table 5.

**Table 5.** Association of AIM2 gene polymorphism with susceptibility to periodontitis and coronary heart disease and severity using multiple logistic regression model.

	(a) Susceptibility	(b) Model I	(c) Model II
Variable	O.R p value	O.R p value	O.R p value
Age	1.223 0.021 *	1.080 0.084	1.072 0.073
(95% CI)	(1.084 to 2.264)	(0.9131 to 1.037)	(0.823 to 1.062)
Sex	0.7362 0.058 *	0.7829 0.2617	1.041 0.7435
(95% CI)	(0.4358 to 0.9668)	(0.3937 to 1.111)	(0.8141 to 1.388)
BMI	1.382 0.040 *	0.928 0.032 *	0.892 0.038 *
(95% CI)	(0.822 to 132.5)	(0.4722 to 1.826)	(0.532 to 8.813)
Socioeconomic status	8.97 0.046 *	3.27 0.060	2.40 0.081
(95% CI)	(1.62 to 67.4)	(1.23 to 3.03)	(0.76 to 1.92)
Tooth brushing frequency	2.26 0.031 *	2.14 0.052 *	1.78 0.066
(95% CI)	(0.50 to 11.3)	(1.01 to 4.62)	(0.76 to 4.18)
BOP	0.537 0.035 *	0.872 0.051 *	1.042 0.001 **
(95% CI)	(0.239 to 0.832)	(0.754 to 1.942)	(1.066 to 1.124)
PLI	0.832 0.025 *	0.9515 0.0308 *	0.903 0.883 *
(95% CI)	(0.723 to 0.943)	(0.4273 to 1.112)	(0.862 to 1.139)
PPD	1.224 0.046 *	1.467 0.026 *	1.138 0.0001 **
(95% CI)	(0.642 to 2.346)	(0.762 to 2.801)	(1.062 to 1.178)
CAL	0.828 0.024 *	0.726 0.027 *	0.714 0.001 **
(95% CI)	(0.506 to 1.357)	(0.472 to 1.113)	(0.618 to 0.862)
AIM2 rs2793845 (GT,TT vs. GG)	3.46 0.0015 *	3.281 0.001 **	2.472 0.0146 *
(95% CI)	(1.632 to 7.546)	(1.822 to 6.05)	(1.214 to 5.108)

* Significant (p< 0.05);

** Significant (p<0.01).

(a) All periodontitis and CHD cases are assigned as case status in logistic regression analyses.

(b) Controls are assigned as control status and coronary heart disease with and without periodontitis cases as case status in logistic regression analyses.

(c) Periodontitis are assigned as control status and coronary heart disease with and without periodontitis cases as case status in logistic regression analyses.

## Discussion

The current investigation identified a correlation between genomic variations related to inflammation in individuals and increased vulnerability to developing periodontitis, both with and without coronary heart disease. This was achieved by analyzing certain genotypes that played a role in the progress of both conditions. Additionally, indicators of periodontal disease severity and the AIM2 gene showed a significant association. The results revealed that males were more susceptible to developing these diseases in all groups at a ratio of 80 males and 20 females. In the AIM2 (rs *2793845*) gene, the P, AS-C, and AS-P groups had noticeably greater frequencies of T allele and distribution (GT, TT) genotypes in blood samples than the control group. An analysis of the AIM2 gene’s single nucleotide polymorphism linked the AIM2 gene’s downstream functional area to the occurrence of periodontitis and coronary heart disease through abnormal gene expression and activation of AIM2 proteins and host molecules. This results in the degradation of the periodontium, impaired functioning of endothelial cells, and production of plaques that contribute to the development of atherosclerosis. This SNP influences the process of inflammation through changes in the shift-regulating activity. Therefore, the presence of these abnormal genetic variations in AIM2 was linked to a higher likelihood of developing periodontitis, both with and without coronary heart disease. Marchesan et al. found that the frequency of a specific gene variant (allele) and the distribution of different genetic combinations (genotypes) of the AIM2 gene (specifically SNP rs *1057028*) may have a substantial impact on the development of periodontal diseases. ^22^ In contrast, Figueira et al. observed that individuals with a specific genetic variation (SNP) in the AIM2 gene (rs *1103577*) had a considerably reduced chance of developing pulmonary tuberculosis (PTB) compared to healthy individuals. ^23^ In polymorphisms of the AIM2 gene, the frequency of the T allele appears to be slightly higher than that in Asians and in the global population. In addition, the association of T alleles of the AIM2 SNP with the probability of periodontal diseases and/or coronary heart disease has been assessed, which suggests the validity of this SNP as a putative genetic risk factor for both diseases in the Iraqi population. The Hardy-Weinberg equilibrium of the AIM2 SNP did not deviate and determined the quality of frequencies of genotypes and alleles for each study group. The current study found that periodontal indicators were greater in the AS-P group than in other study groups. The microbiome of plaque secretes a wide range of microbial residues, which in turn accelerates the degradation of periodontium, leading to pocket formation, resorption of bone, and missing teeth by initiating an inflammatory response, which is in agreement with previous studies. ^16^^,^^24^^,^^25^ Research on the Iraqi population has consistently shown that periodontitis is more common in males than females. This may be attributed to variations in hygienic protocols. ^26^^,^^27^ Male individuals exhibit a higher incidence of atherosclerosis than females because of the protective benefits of estrogen hormones in females. Estrogen has numerous influences on fatty acids, oxygen consumption, blood flow, and oxidative characteristics. ^28^^,^^29^ Therefore, the males appear to be more susceptible to periodontitis and CHD in the patient groups. The Pearson’s correlation coefficient results of this study indicate that AIM2 genetic variation is meaningfully associated with indicators of periodontal disease severity in periodontal disease patient groups. The present findings found significant association (p<0.05) of age, sex, BMI, socioeconomic status, tooth brushing frequency, clinical periodontal parameters, and AIM2 gene polymorphism with susceptibility to periodontitis and CHD. One possible explanation for this association is the presence of genetic and inflammatory mechanisms that contribute to the progression of periodontal disease and plaque production in atherosclerosis in cardiovascular disease. Likewise, the previous research on the role of genetics, the assessment of SNPs’ presumed functionality may expose their pathogenic role in inflammatory diseases such as periodontitis and CHD. ^30^^–^^33^ The aberrant inflammatory reactivity, determined in part by genetic variants in the loci CDKN2B-AS1 (ANRIL), PLG, CAMTA1/VAMP3, and VAMP8, could explain in part the epidemiological link between periodontitis and cardiovascular diseases. These shared genetic factors suggested mechanistic links or immunologic commonalities CHD, periodontitis, and inflammation. ^34^^–^^38^ There has been little research on the correlation between inflammasome SNPs in periodontitis and CHD. ^22^ To the best of the authors’ understanding, this is the first research to look at this relationship in depth. Notably, this study has found pleiotropy role of AIM2 SNP between periodontitis and CHD; the same genetic variant has been observed as being associated with both CHD diseases and periodontitis. This is an intriguing finding because a common genetic background for CHD and periodontitis could be interpreted as similarly aberrant host responses during inflammatory processes, which contribute to the pathogenesis of both diseases. The impairment of the regulatory pathways by genetic factors may be a common pathogenic denominator of CHD disease and periodontitis. ^30^^,^^31^^,^^39^^,^^40^ The present study has several limitations. Popular risk variables for periodontitis and CHD, including cigarette use, high blood pressure, diabetes, and obesity, which might potentially influence or enhance the effects of certain genetic mutations, were deliberately eliminated from the present investigation to prevent bias. Nevertheless, in the presence of various polymorphisms, they may be vulnerable to periodontal and coronary heart diseases. This study is unique in its investigation of the genetic underpinnings of periodontal disease with and without CHD. It had an adequate number of participants, which could be comprehended based on the pilot study to support numerous test adjustments and helped avoid false-positive results commonly found in applicant genes looking at research. Additionally, the results of this case-control study can only be used to establish a link and potential genetic vulnerability in the Iraqi population. The longitudinal studies would be more informative in establishing these relationships but it required more time and costly. Thus, further and longitudinal studies are essential to investigate and explore the function of AIM2 gene polymorphisms as the prognostic and therapeutic implications in the progression of periodontal disease and CHD. To determine whether the AIM2 SNP is applicable to other populations, further experiments are necessary. Considering the correlation between AIM2 genetic substitution and disease severity, our findings not only shed light on disease progression but also suggest a novel approach for treating periodontal disease and coronary heart disease.

### Genetic sequences sharing

All genomic sequences of AIM2 gene are recorded in National Centre for Biotechnology Information. The access numbers are: LC741258, LC741260, LC741262, LC741264, LC741266, LC741268, LC741270, LC741272, LC741354, LC741355, LC741356, LC741357, LC741358, LC741359, LC741360 and LC741361; the access numbers are available at insert in the gene bank ( https://www.ncbi.nlm.nih.gov/genbank/).

## Conclusions

The presence of the T allele and (GT, TT) genotypes of the AIM2 gene, which reveal a wide range of effects on several traits, corresponds to increased vulnerability to both periodontitis and CHD. This genetic variation plays a role in the development of both the diseases. The presence of the AIM2 genetic variation might potentially disrupt the control of epigenetic processes. This can lead to the production of abnormal AIM2 polypeptides and increase the release of inflammatory mediators in both periodontitis and atheroma formation. The AIM2 SNP was also found to be significantly correlated with indicators of periodontal disease severity. The association between these ailments can be attributed to the presence of shared inflammation and molecular mechanisms in their development. The results of the current study raise issues regarding the validity of this SNP as a putative inherited risk factor for these illnesses in Iraqi males.

## Author contributions

Concept and design: Zina Ali Daily and Batool Hassan Al-Ghurabi data collection, processing, or interpreting: the authors; writing up the manuscript: Zina Ali Daily; revising the work critically for key ideas and analyses of statistics: Zina Ali Daily and Batool Hassan Al-Ghurabi; supervision: Batool Hassan Al-Ghurabi.

## Ethical approval

The research followed the tenets of the Declaration of Helsinki and its later amendments for human research. The Ethics Committee of college of Dentistry, University of Baghdad approved this study (Ref. 652, 13/09/2022, Project # 652622in 13/09/2022). All participants entered the study after they were received full information about the nature, aims and processes of the study before signing an informed written consent form.

## Patient consent

All participant signed an informed written consent form which approved by the Ethics Committee following provision of detailed information about the study and its purpose in a consecutive series manner.

## Data Availability

Figshare: 1-Data=Zina Ali-Res=F1000 Research.xlsxData for Zina paperAbsent in Melanoma 2 Gene Associated Periodontitis and Coronary Heart Disease, version 2. DOI: https://doi.org/10.6084/m9.figshare.24017892.v2. ^41^ This project contains the following underlying data: The data contains the recording of periodontal parameters (plaque index (PL), bleeding on probing (BOP), probing pocket depth (ppd) and clinical attachment loss (CAL)) for periodontitis and coronary heart disease patients. Data are available under the terms CC0. Figshare: Data. https://doi.org/10.6084/m9.figshare.24100050.v2. ^42^ This project contains the following extended data: The data contains the details of age, sex, body mass index (BMI), Aim2 genotypes, clinical parameters for periodontitis and coronary heart disease patients with genetic variation of AIM2 gene. Data are available under the terms CC0.
