# Supplementary material for: Absent in Melanoma 2 Gene Associated Periodontitis and Coronary Heart Disease
Source: F1000Res. 2025 Jul 14;13:810. Originally published 2024 Jul 17. [Version 2] doi: 10.12688/f1000research.151954.2 (PMC12584620; doi:10.12688/f1000research.151954.2)
Supplement: Peer review history

# Reviewer report: Reviewer response for version 2

**DOI:** 10.5256/f1000research.184412.r412798

**Published:** 2025-10-23

**Authors:** Snophia Suresh

**Affiliations:** 1 Thai Moogambigai Dental College and Hospital, Chennai, Tamilnadu, India

1. In this study predominance of  males limit generalizability to females

 2. Focus on the single SNP limits understanding of the polygenic and multifactorial nature of periodontal disease and CHD

 3.This cross sectional design cannot establish causality or temporal relationships

 4. Potential confounders such as diet and oral microbiome were not addressed

 5. Functional implications of the polymorphism were not experimentally explored limiting  mechanistic insight

 6. Sample limited to Iraqi population, results may not extrapolate globally

Is the work clearly and accurately presented and does it cite the current literature?

Yes

If applicable, is the statistical analysis and its interpretation appropriate?

Yes

Are all the source data underlying the results available to ensure full reproducibility?

Yes

Is the study design appropriate and is the work technically sound?

Yes

Are the conclusions drawn adequately supported by the results?

Yes

Are sufficient details of methods and analysis provided to allow replication by others?

Yes

Reviewer Expertise:

Periodontal medicine

I confirm that I have read this submission and believe that I have an appropriate level of expertise to confirm that it is of an acceptable scientific standard, however I have significant reservations, as outlined above.

# Reviewer report: Reviewer response for version 2

**DOI:** 10.5256/f1000research.184412.r398472

**Published:** 2025-08-13

**Authors:** Athraa Ali Mahmood

**Affiliations:** 1 Department of Oral Surgery and Periodontics, Mustansiriyah University, Baghdad, Iraq

Thank you for the opportunity to review the revised manuscript and the author responses. I have reviewed the updated version and have no further comments to make. I am satisfied with the revisions and the clarifications provided by the authors.

 Sincerely,

 [Dr. Athraa Ali Mahmood]

Is the work clearly and accurately presented and does it cite the current literature?

Yes

If applicable, is the statistical analysis and its interpretation appropriate?

Yes

Are all the source data underlying the results available to ensure full reproducibility?

Yes

Is the study design appropriate and is the work technically sound?

Yes

Are the conclusions drawn adequately supported by the results?

Yes

Are sufficient details of methods and analysis provided to allow replication by others?

Yes

Reviewer Expertise:

NA

I confirm that I have read this submission and believe that I have an appropriate level of expertise to confirm that it is of an acceptable scientific standard.

# Reviewer report: Reviewer response for version 1

**DOI:** 10.5256/f1000research.166650.r304567

**Published:** 2024-07-30

**Authors:** Paras Ahmad

**Affiliations:** 1 College of Dentistry, University of Saskatchewan, Saskatoon, Saskatchewan, Canada

The study on AIM2 gene polymorphisms and their association with periodontal disease and coronary heart disease (CHD) presents an interesting case-control design.

 However, several deficiencies in the methods can be identified:

- It is not clear how well the control group (healthy participants) is matched with the other groups in terms of age, gender, and other potential confounding factors. Proper matching is crucial to ensure that differences observed are due to the variables of interest ( *AIM2* gene polymorphisms) rather than other extraneous variables.
- The clinical assessments of periodontal disease indicators and CHD diagnosis involve subjective components and specialist interpretations. There could be variability in these assessments, and the study does not mention blinding of assessors, which could introduce bias.
- The study relies on one-way ANOVA and Odds/Ratios without detailed reporting of how these analyses were conducted, including handling potential confounders. Additionally, the use of Pearson correlation coefficients for genetic polymorphism analysis might not be the most appropriate method, given the typically non-linear relationships and categorical nature of genetic data.
- While the study attempts to control for some confounders by excluding certain participants, it does not appear to account for other potential confounders, such as socioeconomic status, diet, and oral hygiene practices, which could influence both periodontal health and CHD.
- The focus on a single gene polymorphism (AIM2 G/T rs2793845) might be too narrow, as complex diseases like periodontal disease and CHD are often influenced by multiple genetic and environmental factors. Additionally, the study does not discuss the functional implications of this polymorphism or its interaction with other genetic factors.
- The study design is cross-sectional, limiting the ability to infer causation or understand the temporal relationship between AIM2 polymorphisms, periodontal disease, and CHD. Longitudinal studies would be more informative in establishing these relationships.

Addressing these deficiencies would enhance the study’s rigor, reliability, and applicability.

 Thanks

Is the work clearly and accurately presented and does it cite the current literature?

Partly

If applicable, is the statistical analysis and its interpretation appropriate?

No

Are all the source data underlying the results available to ensure full reproducibility?

Partly

Is the study design appropriate and is the work technically sound?

No

Are the conclusions drawn adequately supported by the results?

No

Are sufficient details of methods and analysis provided to allow replication by others?

Partly

Reviewer Expertise:

Periodontal diseases; periodontitis; gingivitis; saliva; proteins; genes; proteomics; mass spectrometry

I confirm that I have read this submission and believe that I have an appropriate level of expertise to confirm that it is of an acceptable scientific standard, however I have significant reservations, as outlined above.

# Response

**Published:** 2025-07-05

**Authors:** Zina Daily

**Affiliations:** Department of Periodontics, University of Al-Ameed, Baghdad, Iraq

Thank you for your letter and for the reviewer’ comments concerning our manuscript. Those comments are all valuable and very helpful for revising and improving our paper, as well as the important guiding significance to our research. We have studied comments carefully and have made correction which we hope meet with approval.

**Response to comment of reviewer2**

 1.Response to comment: 1- It is not clear how well the control group (healthy participants) is matched with the other groups in terms of age, gender, and other potential confounding factors. Proper matching is crucial to ensure that differences observed are due to the variables of interest (AIM2 gene polymorphisms) rather than other extraneous variables.

 Response: Thank you for your suggestion. I agree that the information of control group (healthy participants) and the other groups in terms of age, gender, and other potential confounding factors are due to the variables of interest (AIM2 gene polymorphisms), so that we matched the same range of subjects to avoid bias of the study and focusing on one variable (genetic changes) effecting and inflammatory process. We added the socioeconomic status, tooth brushing frequency at least two times per- day in the variable because these were recorded during data collection.

 2.Response to comment: 2-The clinical assessments of periodontal disease indicators and CHD diagnosis involve subjective components and specialist interpretations. There could be variability in these assessments, and the study does not mention blinding of assessors, which could introduce bias.

 Response: Thank you for pointing that out. We measured standardized periodontal clinical indicators to detect healthy periodontium and periodontal disease, while specialist of cardiology is blinding to subjects of CHD patients that related to CHD with periodontitis group or CHD with healthy periodontium group. As well as, the blind laboratory doctor collected, process blood samples and detected gene polymorphism. The statistic of data process by blind analyzer. We will revise the material and methods (Sample) to make this part clearer and more explicit.

 3.Response to comment: 3-The study relies on one-way ANOVA and Odds/Ratios without detailed reporting of how these analyses were conducted, including handling potential confounders. Additionally, the use of Pearson correlation coefficients for genetic polymorphism analysis might not be the most appropriate method, given the typically non-linear relationships and categorical nature of genetic data.

 Response Thank you for your question. I was adding detailed reporting of how these analyses were conducted in revision. Additionally, the use of multiple logistic regression for analysis the association of AIM2 gene polymorphism with susceptibility to periodontitis and CHD in Table 5.

 4- Response to comment: 4-While the study attempts to control for some confounders by excluding certain participants, it does not appear to account for other potential confounders, such as socioeconomic status, diet, and oral hygiene practices, which could influence both periodontal health and CHD.

 Response: Thank you for your question. I was adding the account for other potential confounders, such as socioeconomic status, and oral hygiene practices in the new revision, which could influence both periodontal health and CHD in table 1.

 5- Response to comment: 5-The focus on a single gene polymorphism (AIM2 G/T rs2793845) might be too narrow, as complex diseases like periodontal disease and CHD are often influenced by multiple genetic and environmental factors. Additionally, the study does not discuss the functional implications of this polymorphism or its interaction with other genetic factors.

 Response: Thank you for your question. In our study, we examined Iraqi subjects concerning the SNPs of AIM2 gene which detected the only single nucleotide polymorphism in Aim2 gene at location rs2793845. It was a significant difference between healthy individuals, periodontal disease and CHD in the examined groups of Iraqi subjects. Furthermore, the functional implications of this polymorphism were added and explained in discussion part.

 6- Response to comment: 6-The study design is cross-sectional, limiting the ability to infer causation or understand the temporal relationship between AIM2 polymorphisms, periodontal disease, and CHD. Longitudinal studies would be more informative in establishing these relationships.

 Response： Thank you for this suggestion. The longitudinal studies would be more informative in establishing these relationships, but it required more time and costly. Thus, further and longitudinal studies are essential to investigate and explore the function of AIM2 gene polymorphisms as the prognostic and therapeutic implications in the progression of periodontal disease and CHD.

# Reviewer report: Reviewer response for version 1

**DOI:** 10.5256/f1000research.166650.r304564

**Published:** 2024-07-24

**Authors:** Athraa Ali Mahmood

**Affiliations:** 1 Department of Oral Surgery and Periodontics, Mustansiriyah University, Baghdad, Iraq

Present the work clearly and accurately. However, it is better to add a few words about the statistical method within the methods of the abstract and add the value of p in the results of the abstract for further clarification.

 It is also preferable to add a source to an existing paragraph within the article and explain the reason for that (after assessing five patients with periodontal disease, two calibrating meetings for indicators of periodontal disease severity were obtained within an hour).

 Sometimes there are many words that have been abbreviated in the research, so it would be preferable to standardize the abbreviation and not mention the whole word again, for example, body mass index (BMI).

Is the work clearly and accurately presented and does it cite the current literature?

Yes

If applicable, is the statistical analysis and its interpretation appropriate?

Yes

Are all the source data underlying the results available to ensure full reproducibility?

Yes

Is the study design appropriate and is the work technically sound?

Yes

Are the conclusions drawn adequately supported by the results?

Yes

Are sufficient details of methods and analysis provided to allow replication by others?

Yes

Reviewer Expertise:

NA

I confirm that I have read this submission and believe that I have an appropriate level of expertise to confirm that it is of an acceptable scientific standard.

# Response

**Published:** 2025-07-05

**Authors:** Zina Daily

**Affiliations:** Department of Periodontics, University of Al-Ameed, Baghdad, Iraq

Thank you for your letter and for the reviewer’ comments concerning our manuscript. Those comments are all valuable and very helpful for revising and improving our paper **Response to comment of reviewer1**

 1.Response to comment: 1- It is better to add a few words about the statistical method within the methods of the abstract and add the value of p in the results of the abstract for further clarification.

 Response: Thank you for your valuable feedback. I will revise the abstract to include a clear sentence and add the value of p in the results of the abstract for further clarification.

 2.Response to comment: 2-It is also preferable to add a source to an existing paragraph within the article and explain the reason for that (after assessing five patients with periodontal disease, two calibrating meetings for indicators of periodontal disease severity were obtained within an hour).

 Response: Thank you for your suggestion. I agree that the explaining of reason for that (after assessing five patients with periodontal disease, two calibrating meetings for indicators of periodontal disease severity were obtained within an hour) should be explicitly stated for clarity. I am explaining this work and add a source to an existing paragraph within the article.

 3.Response to comment:3-Sometimes there are many words that have been abbreviated in the research, so it would be preferable to standardize the abbreviation and not mention the whole word again, for example, body mass index (BMI).

 Response: Thank you for your comment. We standardize the abbreviation of body mass index (BMI) and other abbreviation.
